# Significantly increased expression of OCT4 and ABCG2 in spheroid body-forming cells of the human gastric cancer MKN-45 cell line

**DOI:** 10.3892/ol.2013.1506

**Published:** 2013-08-01

**Authors:** JIANMING LIU, LEI WANG, LILIN MA, JUNFEI XU, CHUN LIU, JIANGUO ZHANG, JIE LIU, RUIXIN CHEN

**Affiliations:** 1 Department of General Surgery, Affiliated Hospital of Nantong University, Nantong, Jiangsu 226001, P.R. China; 2 Nantong Egens Biotechnology Co., Ltd., Nantong, Jiangsu 226010, P.R. China; 3 Laboratory Animal Center, Nantong University, Nantong, Jiangsu 226001, P.R. China; 4 Department of Pathology, Affiliated Hospital of Nantong University, Nantong, Jiangsu 226001, P.R. China; 5 Research Center of Hand Surgery, Affiliated Hospital of Nantong University, Nantong, Jiangsu 226001, P.R. China

**Keywords:** human gastric cancer, cancer stem cell, OCT4, ABCG2

## Abstract

The cancer stem cell (CSC) theory hypothesizes that CSCs are the cause of tumor formation, recurrence and metastasis. Key to the study of CSCs is their isolation and identification. The present study investigated whether spheroid body-forming cells in the human gastric cancer (GC) MKN-45 cell line are enriched for CSC properties, and also assessed the expression of the candidate CSC markers, octamer-binding transcription factor-4 (OCT4) and adenosine triphosphate-binding cassette transporter G2 (ABCG2) in the MKN-45 spheroid body cells. The MKN-45 cells were plated in a stem cell-conditioned culture system to allow for spheroid body formation. The expression levels of OCT4 and ABCG2 in the spheroid body cells were assessed by qPCR, western blot analysis and immunofluorescence staining, while the tumorigenicity of the spheroid body-forming cells was assessed by *in vivo* xenograft studies in nude mice. The MKN-45 cells were able to form spheroid bodies when cultured in stem cell-conditioned medium. The spheroid body-forming cells showed a significantly higher (P<0.01) expression of OCT4 and ABCG2 compared with the parental cells. These data suggest that the spheroid body cells from the MKN-45 GC cell line cultured in stem cell-conditioned medium possessed gastric CSC properties. The co-expression of OCT4 and ABCG2 by these cells may represent the presence of a subpopulation of gastric CSCs.

## Introduction

Globally, gastric cancer (GC) is the fourth most common malignancy among all types of cancers, and mortality due to GC is second only to lung cancer (1). Of all GC cases, >70% occur in developing countries and half the world total occurs in Eastern Asia (mainly China) (2). Despite improvements in surgical techniques and the development of new chemotherapeutic regimens, the results are often disappointing. The overall five-year survival rate for patients who undergo curative surgical resections for GC ranges between 47.0 and 60.4% (3). Although a number of studies have investigated the pathogenesis of the disease, the true mechanisms of GC carcinogenesis remain obscure (3).

In the past few years, it has been hypothesized that tumors are most likely initiated by a minority of cells, known as cancer stem cells (CSCs) (4). According to the American Association for Cancer Research (AACR), CSCs are defined as subpopulations of cells within a tumor that possess the capacity for self-renewal and cause the heterogeneous lineage of cancer cells that constitute the tumor (5). The difficulty in eradicating tumors may be due to the fact that conventional treatments target the bulk of the tumor cells, leaving the CSCs, which are involved in tumor maintenance, therapy resistance, tumor progression, recurrence and distant metastasis, unaffected. According to this hypothesis, identifying and eradicating CSCs may be an effective treatment modality (6).

The question of how to isolate and identify CSCs is key to the research into them. Three distinct methodologies based on the properties of CSCs have been used successfully for the isolation of these cells from solid tumors (5,7,8). The first is fluorescence-activated cell sorting (FACS) according to CSC-specific cell surface markers, such as cluster of differentiation 44 (CD44) or CD133 (8,9). The second is that side populations (SP) of tumor cells, which exhibit intracellular Hoechst 33342 exclusion *in vitro* and also preferentially express adenosine triphosphate-binding cassette transporter G2 (ABCG2) (10–12), are isolated and characterized as CSCs (13–15). The third is the spheroid body formation assay in which cells are cultured in non-adherent conditions in a serum-free medium supplemented with basic fibroblast growth factor (bFGF) and epidermal growth factor (EGF). The latter approach has been suggested as a practical approach for individual solid tumor tissues or cancer cells (16,17). The present study aimed to develop spheroid body-forming cells in the MKN-45 GC cell line and to analyze the expression of two putative candidate stem cell markers, octamer-binding transcription factor-4 (OCT4) and ABCG2, in spheroid body-forming cells.

## Materials and methods

### Culture of parental cells and spheroid body-forming cells

The human MKN-45 GC cell line was purchased from the Cell Bank of the Chinese Academy of Sciences (Shanghai, China) and cultured in RPMI-1640 medium containing 10% fetal bovine serum (FBS), then plated at a density of 1×10^6^ live cells per 75-cm^2^ flask. Once the cells had become attached they were subsequently passaged upon confluence. Spheroid bodies were derived by placing the parental cells into serum-free RPMI-1640 culture medium containing 1% N-2 supplement, 2% B-27 supplement (both Invitrogen, Carlsbad, CA, USA), 1% antibiotic mixture (Gibco, Carlsbad, CA, USA), 20 ng/ml human FGF-2 and 100 ng/ml EGF (both Chemicon, Temecula, CA, USA). The parental cells were plated in 96-well ultra-low attachment plates (Corning Inc., Corning, NY, USA) at 100 cells per well. Two weeks later, the plates were analyzed for spheroid body formation and quantified using an inverted microscope (Olympus, Tokyo, Japan) at ×40 and ×100 magnification. Once the primary spheroid bodies had reached a size of ~200–500 cells per spheroid body, they were dissociated at a density of 1,000 cells per ml and 100 μl single cell suspension was seeded in each well of the 96-well ultra-low attachment plates (Corning) in serum-free medium, as described previously. Two weeks later, the wells were analyzed for subspheroid body formation.

### qPCR

Total RNA was extracted from the parental and spheroid body-forming cells using Qiagen RNeasy mini kits (Qiagen, Hilden, Germany) according to the manufacturer's instructions. RNA was treated with DNase I (Qiagen) to eliminate genomic DNA contamination. The integrity and purification of the RNA samples were monitored by agarose gel electrophoresis. The concentration of RNA was determined by repeated OD measurements of aliquots at a wavelength of 260 nm. A reverse-transcription reaction to transcribe 1 μg total RNA into complementary DNA was performed using reagents of an Omniscript RT kit (Qiagen).

To determine the fold changes in the expression of each gene, qPCR was performed using an Eppendorf Mastercycler^®^ ep realplex (2S; Eppendorf, Hamburg, Germany). EvaGreen^®^ (Biotium Inc., Hayward, CA, USA) served as a dye that bound to the amplified DNA to emit fluorescence during the reactions. EvaGreen has emerged as an optimal green fluorescent DNA dye for qPCR, of equal or better sensitivity compared with SYBR Green I (18). The 25-μl reaction mixture contained 12.5 μl Evagreen qPCR Master Mix (Biotium Inc.), 1 μl primers (10 mM), 1 μl template cDNA and 10.5 μl double distilled water (ddH_2_O). The glyceraldehyde-3 phosphate dehydrogenase (GAPDH) gene served as an internal control for the expression levels of the target apoptosis genes. The primer sequences are shown in Table I. After an initial incubation for 2 min at 96°C, the reactions were performed for 40 cycles of 96°C for 15 sec and 60°C for 45 sec (florescence collection). Fluorescence was measured during the extension step of each cycle. A melting curve analysis was performed to ensure the amplification of a single PCR product. Reactions with no template were included as a negative control. By setting the threshold at the level of the middle steady portion of the reaction cycles versus florescence curve, the Ct values of the target genes were calculated using Mastercycler ep realplex analysis software (Eppendorf) and the 2^−ΔΔCT^ method. Finally, the PCR products were separated by 1.5% agarose gel electrophoresis in the presence of ethidium bromide, prior to being visualized on an ultraviolet illuminator to verify product sizes and then recorded. qPCR was performed independently three times in triplicate.

### Immunofluorescence staining for candidate CSC markers

In brief, the cells plated onto poly-L-lysine-coated glass coverslips were fixed with 4% paraformaldehyde, then washed with phosphate-buffered saline (PBS). The cells were permeabilized with 0.1% Triton X-100/PBS for 10 min and subsequently incubated with primary antibodies (anti-OCT4 rabbit polyclonal and anti-ABCG2 mouse monoclonal antibodies; Santa Cruz Biotechnology, Inc., Santa Cruz, CA, USA). The cells were further probed with fluorescein isothiocyanate or rhodamine-tagged secondary antibodies. 4′,6-Diamidino-2-phenylindole (DAPI), which is a fluorescent stain that binds strongly to A-T rich regions in DNA, was used for the nuclear counterstain. The fluorescence was recorded using an inverted fluorescence microscope (Leica, Mannheim, Germany).

### Western blot analysis

For the western blot analyses, proteins were harvested from the cells plated to between 70 and 80% confluence. Spheroid body-forming or parental cells were lysed directly in lysis buffer to collect whole cell extracts. Protein samples for western blotting were prepared by boiling the cell extracts following the addition of denaturing sample buffer. Subsequently, the proteins were separated using SDS-PAGE on an 8 or 15% gel, then transferred onto PVDF membranes. The membranes were incubated at 4°C overnight with primary antibody and subsequently incubated with horseradish peroxidase-conjugated secondary antibodies for 1 h at room temperature. Finally, protein bands were visualized using chemiluminescence (Santa Cruz) exposure on BioMax film (Kodak, Rochester, NY, USA). A 1:200 concentration was used for the anti-OCT4 and anti-ABCG2 primary antibodies (Santa Cruz Biotechnology, Inc.).

### In vivo tumorigenicity experiments

Male, six to eight-week-old athymic nude mice (nu/nu) were obtained from the Shanghai Laboratory Animal Center of the Chinese Academy of Sciences (Shanghai, China) and housed under pathogen-free conditions in a barrier animal facility. All animal procedures were performed with the approval of the Animal Ethics Committee of Nantong University.

For the *in vivo* tumorigenicity experiments, equal numbers (1×10^4^, 2×10^4^, 2×10^5^ and 2×10^6^) of freshly dissociated cells were suspended in 200 μl PBS and then the spheroid body-forming cells were injected subcutaneously into the right rear flank of each mouse (six mice per group). The parental cells were injected subcutaneously into the left rear flank of each mouse and the tumorigenic capacity of the spheroid body-forming and parental cells was evaluated. The mice were observed for tumor growth every 10 days over eight weeks, then sacrificed by cervical dislocation. The grafts were removed, fixed with 10% buffered formalin and stained with hematoxylin and eosin.

### Statistical analysis

All experiments were repeated at least three times and representative results are presented. All values in the figures and text are shown as the mean ± SD. Statistical analyses were performed using the SPSS statistical software package (SPSS/PC+; SPSS Inc., Chicago, IL, USA). Significant differences among mean values were evaluated by Student's t-test. A two-sided value of P<0.05 was considered to indicate a significant difference.

## Results

### GC cells form anchorage-independent spheroid bodies

**Figure 1:**
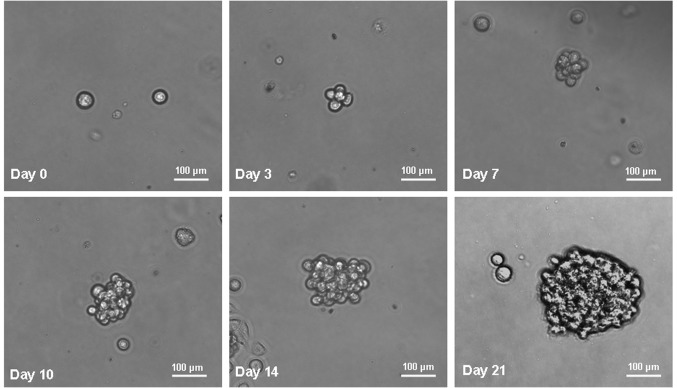
Phase images of a single MKN-45 spheroid body-derived cell cultured in a 96-well ultra-low attachment plate under anchorage-independent, serum-free conditions. The propagation of a single cell was recorded separately at days 0, 3, 7, 10, 14 and 21.

MKN-45 parental cells were cultured in serum-free medium as described in the methods section. Under these conditions, the cells grew in non-adherent, three-dimensional spheroid clusters known as spheroid bodies. The self-renewing capacity of these spheroid body-forming cells was assessed by dissociation into single cells and growth in serum-free medium as described in the methods section. The spheroid bodies appeared to be taking shape at day 3. At day 7, the spheroid bodies were formed substantially. At day 10 and 14, the spheroid bodies were completely formed. At day 21, the spheroid bodies had become well-rounded structures composed of numerous, compacted cells. Fig. 1 shows the generation of a spheroid body from a single MKN-45 cell. The propagation of a single cell cultured in a 96-well dish was recorded separately at days 0, 3, 7, 10, 14 and 21.

### Spheroid body-forming cells overexpress candidate CSC markers, OCT4 and ABCG2

**Figure 2:**
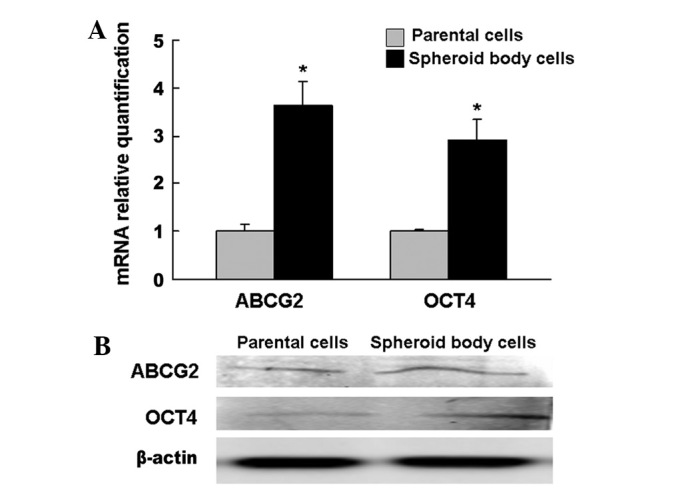
Spheroid body-forming cells overexpress the candidate cancer stem cell (CSC) markers, OCT4 and ABCG2. (A) qPCR analysis demonstrating the elevated expression of OCT4 and ABCG2 genes in the MKN-45 spheroid body-forming cells compared with the parental cells (^*^P<0.01). (B) Western blotting analysis showing the elevated expression of OCT4 and ABCG2 proteins in the MKN-45 spheroid body-forming cells compared with the parental cells. OCT4, octamer-binding transcription factor-4; ABCG2, adenosine triphosphate-binding cassette transporter G2.

qPCR and western blotting were performed on the spheroid body-forming and parental cells. The results showed that significantly more cells expressed OCT4 and ABCG2 among the spheroid body-forming cells compared with the parental cells (Fig. 2).

### Intracellular localization of OCT4 and ABCG2 in spheroid body-forming cells

**Figure 3:**
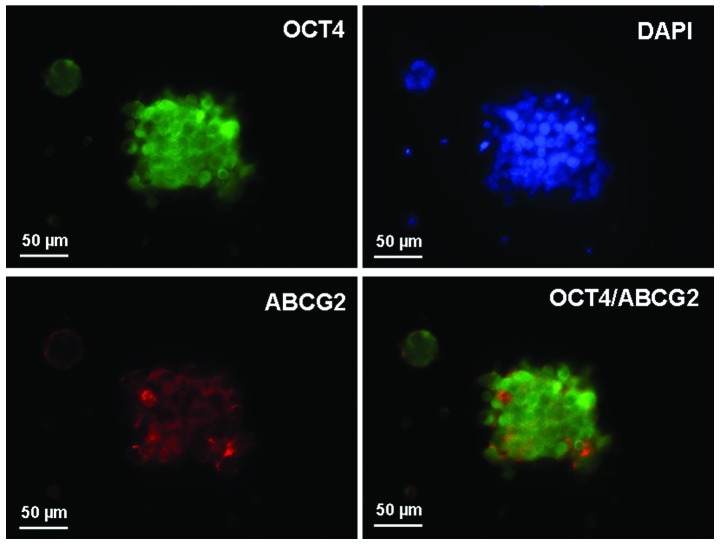
Intracellular localization of OCT4 and ABCG2 by immunofluorescence staining. Dual staining of OCT4 and ABCG2 indicated that OCT4-positive cells were co-stained with ABCG2. 4′6-Diamidino-2-phenylindole (DAPI) was used for the nuclear counterstain. OCT4, octamer-binding transcription factor-4; ABCG2, adenosine triphosphate-binding cassette transporter G2.

To examine the subcellular localization of OCT4 and ABCG2 in the spheroid body-forming cells, immunofluorescence staining of OCT4 and ABCG2 was performed. Positive staining for OCT4 and ABCG2 was observed, with OCT-4 mainly present within the perinuclear cytoplasm of the spheroid body-forming cells and ABCG2 mainly present in the membrane. Dual staining for OCT4 and ABCG2 indicated that the candidate CSC markers, OCT4 and ABCG2, were colocalized in the spheroid body-forming cells (Fig. 3).

### Spheroid body-forming cells exhibit high tumorigenicity in vivo

**Figure 4:**
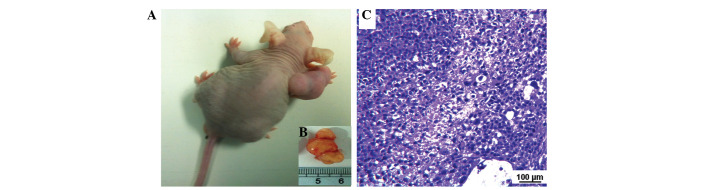
Spheroid body-forming cells exhibit high tumorigenicity *in vivo.* (A) Representative example showing a xenograft tumor formed after subcutaneous injection with 2×10^4^ MKN-45 spheroid body-forming cells. (B) Nodule formed by the injection of 2×10^4^ MKN-45 spheroid body-forming cells. (C) Hematoxylin and eosin staining confirming that the histological features of the xenograft tumors induced by the MKN-45 spheroid body-forming cells were those of gastric cancer (GC).

The tumorigenicity experiments *in vivo* showed that as few as 2×10^4^ cells from an MKN-45 spheroid body were able to form a tumor when subcutaneously injected into nude mice (Fig. 4A and B), while 2×10^6^ parental cells were required for the same effect. This value was 100-fold higher than that for the spheroid body-forming cells. Moreover, the spheroid body-forming cells generated subcutaneous tumors with larger volumes in shorter times compared with those generated from the parental cells. The transplanted tumors were confirmed as GC using hematoxylin and eosin staining (Fig. 4C).

## Discussion

OCT-4, a member of the POU-domain transcription factor family, is expressed in pluripotent embryonic stem and germ cells (19). OCT-4 functions as a master switch during differentiation by regulating the pluripotent potential in stem cells (20–22). The expression of OCT-4 has also been shown in human breast cancer stem-like cells and its expression may be implicated in self-renewal and tumorigenesis (23). The ABCG2 transporter is a member of the ATP-binding cassette transporter family responsible for the SP phenotype in various human cancers and the corresponding non-malignant tissues, and is widely used to detect and isolate somatic stem/progenitor cells (24). Fukuda *et al* demonstrated that the SP fraction of GC cells had a sphere forming ability and high tumorigenicity in non-obese diabetic mice, as well as resistance to anticancer drugs and an immunophenotype similar to that of stem cells (25).

Previously, Chen *et al* demonstrated that OCT-4 siRNA treatment resulted in a significant downregulation of ABCG2 expression and an increase in the chemosensitivity of CD133-positive cells (26). Jia *et al* enriched CD90^+^/CD133^+^ hepatocellular carcinoma CSCs using spheroid body formation and observed that OCT4 and ABCG2 were highly expressed in the enriched CD90^+^/CD133^+^ liver CSCs and were closely associated with chemotherapy drug resistance (27). However, the expression of OCT4 and ABCG2 has not been reported in the spheroid body-forming cells of GC.

Spheroid body cultures have increasingly been used as a method for enriching stem cells, which relies on their property of anchorage-independent growth. Through the application of a spheroid body culture, numerous types of potential CSC subpopulations have been reported to have been isolated and enriched from primary tumors (28–35). The spheroid body-forming cells from primary tumors, including those of ovarian and breast cancer, have demonstrated stem-like properties and expressed their CSC markers (29,33). To the best of our knowledge, there have been few reports on the isolation and characterization of gastric CSCs by the method of spheroid body culture. Consequently, the present study developed spheroid body cells by cultivating the human MKN-45 GC cell line within a defined serum-free medium, and demonstrated that the cells derived from the spheroid bodies were able to generate greater numbers of new spheroid bodies and subcutaneous tumors in nude mice, with larger volumes in shorter times, compared with those generated from the parental cells. This indicated that the spheroid body-forming cells were capable of self-renewal and proliferation and possessed higher tumorigenicity, which are key characteristics of CSCs.

To further investigate the CSC properties of spheroid body-forming cells, the MKN-45 spheroid body-forming cells were evaluated for the expression of the putative candidate CSC markers, OCT4 and ABCG2. The present study observed that OCT4 and ABCG2 were overexpressed in the MKN-45 spheroid body-forming cells compared with the parental cells. More significantly, a focus was first placed on the question of whether there is a physical linkage between OCT4 and ABCG2 in spheroid body-forming cells. The OCT4-positive spheroid body-forming cells were observed to be co-stained with ABCG2, indicating the co-expression of OCT4 and ABCG2 in MKN-45 spheroid bodies, which may represent a subpopulation of gastric CSCs.

In conclusion, the study demonstrated that non-adherent spheroid body-forming cells from the human MKN-45 GC cell line that are cultured in a defined serum-free medium possess gastric CSC properties. Since these cells co-expressed OCT4 and ABCG2 in the MKN-45 spheroid bodies, they may represent a subpopulation of gastric CSCs. The correlation between OCT4 and ABCG2 in GC stem cells requires further investigation.

## Figures and Tables

**Table I:** Base sequences of primers for qPCR.

Primer name	Sequence
OCT4-Forward	AACGACCATCTGCCGCT
OCT4-Reverse	CGATACTGGTTCGCTTTCTCT
ABCG2-Forward	TGAGGGTTTGGAACTGTGG
ABCG2-Reverse	GATTCTGACGCACACCTGG
GAPDH-Forward	GGCATCCTGGGCTACACT
GAPDH-Reverse	CCACCACCCTGTTGCTGT

OCT4, octamer-binding transcription factor-4; ABCG2, adenosine triphosphate-binding cassette transporter G2.

## Abbreviations

CSC: cancer stem cell
GC: gastric cancer
OCT4: octamer-binding transcription factor-4
ABCG2: adenosine triphosphate binding cassette transporter G2
